# C-reactive protein elevation predicts in-hospital deterioration after aneurysmal subarachnoid hemorrhage: a retrospective observational study

**DOI:** 10.1007/s00701-022-05256-0

**Published:** 2022-05-26

**Authors:** Ostini Alessandro, Warschkow Rene, Wolf Stefan, Filipovic Miodrag, Seule Martin, Bozinov Oliver, Pietsch Urs

**Affiliations:** 1 Department of Intensive Care Medicine, Inselspital, Bern University Hospital, CH-3010 Bern, Switzerland; 2 Department of Neurosurgery, Kantonsspital St. Gallen, St. Gallen, Switzerland; 3 Department of Surgery, Kantonsspital St. Gallen, St. Gallen, Switzerland; 4 Institute of Medical Biometry and Informatics, University of Heidelberg, Heidelberg, Germany; 5 Department of Neurosurgery, Charité Campus Mitte, Charité-Universitätsmedizin Berlin, Berlin, Germany; 6 Division of Anaesthesiology, Intensive Care, Rescue and Pain Medicine, Kantonsspital St. Gallen, St. Gallen, Switzerland; 7 Department of Emergency Medicine, Inselspital, Bern University Hospital, University of Bern, Bern, Switzerland

**Keywords:** Subarachnoid hemorrhage, C-reactive protein, Delayed cerebral ischemia

## Abstract

**Background:**

There is increasing evidence that inflammation plays a role in the pathogenesis of aneurysmal subarachnoid hemorrhage (aSAH) and in the development of delayed cerebral ischemia (DCI). However, the assessment and interpretation of classically defined inflammatory parameters is difficult in aSAH patients. The objective of this study was to investigate the relationship between easily assessable findings (hyperventilation, fever, white blood cell count (WBC), and C-reactive protein (CRP)) and the occurrence of DCI and unfavorable neurological outcome at discharge in aSAH patients.

**Methods:**

Retrospective analysis of prospectively collected data from a single center cohort. We evaluated the potential of clinical signs of inflammation (hyperventilation, fever) and simple inflammatory laboratory parameters CRP and WBC to predict unfavorable outcomes at discharge and DCI in a multivariate analysis. A cutoff value for CRP was calculated by Youden’s *J* statistic. Outcome was measured using the modified Rankin score at discharge, with an unfavorable outcome defined as modified Rankin scale (mRS) > 3.

**Results:**

We included 97 consecutive aSAH patients (63 females, 34 males, mean age 58 years) in the analysis. Twenty-one (22%) had major disability or died by the time of hospital discharge. Among inflammatory parameters, CRP over 100 mg/dl on day 2 was an independent predictor for worse neurological outcome at discharge. The average C-reactive protein level in the first 14 days was higher in patients with a worse neurological outcome (96.6, SD 48.3 vs 56.3 mg/dl, SD 28.6) in the first 14 days after aSAH. C-reactive protein on day 2 was an indicator of worse neurological outcome. No inflammatory parameter was an independent predictor of DCI. After multivariate adjustment, DCI, increased age, and more than 1 day of mechanical ventilation were significant predictors of worse neurological outcome.

**Conclusions:**

Early elevated CRP levels were a significant predictor of worse neurological outcome at hospital discharge and may be a useful marker of later deterioration in aSAH.

## Background

Aneurysmal subarachnoid hemorrhage (aSAH) is a medical emergency associated with substantial long-term morbidity and high mortality [4]. Prediction of functional neurological outcome plays a crucial role in the management of patients affected by this condition. In traditional prediction models, the most common variables associated with an unfavorable neurological outcome are older age, poor neurological status on admission (World Federation of Neurological Surgeons (WFNS) or Hunt and Hess scale), and significant volume of blood on admission CT scan (Fisher grade). However, neurological status is often confounded in unconscious and sedated patients by acute complications such as occlusive hydrocephalus or intracerebral hematoma [1, 3].

Because of emerging evidence of the role of systemic and compartmental inflammation in the pathogenesis of aSAH and in the development of delayed cerebral ischemia (DCI) with or without presence of cerebral vasospasm (CVS), increasing attention is being paid to the relationship between clinical signs of systemic inflammation such as the systemic inflammatory response (SIRS) and neurological outcome in aSAH [5, 16]. In particular, an association between hyperventilation [19, 26, 27], fever [7, 12], and a high white blood cell count (WBC) [18, 20] and unfavorable neurological outcome after aSAH has been described. Moreover, high levels of interleukin 6 (IL-6) and C-reactive protein (CRP) in cerebrospinal fluid (CSF) have been associated with secondary ischemic complications after aSAH, leading to an unfavorable outcome [8, 9, 17]. Serum IL-6 levels have also been associated with DCI and worse outcome [9, 14], although results of studies on serum CRP levels are conflicting [6, 8, 22].

The assessment and interpretation of classically defined and studied parameters of SIRS can be difficult in a clinical setting with severely ill aSAH patients. Catecholamines may be used to induce hypertension to treat CVS-associated DCI; active temperature management may mask fever while the measurement of parameters such as IL-6 may not be widely available, especially in CSF probes, making their use in clinical practice problematic.

The aim of the present study was to investigate the relationship between easily assessable clinical findings (hyperventilation, fever, WBC, and CRP) and the occurrence of DCI, as well as functional neurological outcome in aSAH patients.

## Methods

This retrospective analysis of prospectively collected data was performed at a single center. Ethical approval was obtained from the local institutional review board (protocol no. EKSG 12/016). Reporting was done according to the STROBE guidelines (Strengthening the Reporting of Observational Studies in Epidemiology).

The study included patients aged 18 years and above, who were consecutively admitted to the ICU with a confirmed diagnosis of aSAH between 1 January 2014 and 31 December 2018. All patients with aneurysm occlusion using either endovascular coiling or microsurgical clipping were included. Patients with non-aneurysmatic SAH and patients who did not undergo aneurysm closure were excluded from the analysis.

The clinical severity at the time of admission was assessed by the neurosurgeon on duty according to the WFNS scale and divided into two groups (low grade (WFNS 1–3) and high grade (WFNS 4–5)). Blood volume on the initial computed tomography (CT) scan was assessed by Fisher grade and also categorized into two groups (Fisher 1–2 and 3–4). The modified Rankin scale (mRS) was used to evaluate neurological outcome at hospital discharge.

### ICU management of SAH and definitions

All aSAH patients were treated according to the local SAH management guidelines consistent with the Guidelines for the Management of Aneurysmal Subarachnoid Hemorrhage by the American Heart Association/American Stroke Association [4].

In addition to general intensive care, all patients were monitored for clinical deterioration. Transcranial Doppler sonography to measure cerebral blood flow velocity in the basal cerebral arteries as a predictor of CVS was performed daily. In cases of new onset neurological deficits or a significant increase in Doppler velocity, CT angiography (CTA) was performed to confirm or exclude the presence of arterial vasospasm. On day 7, a screening CTA was performed in all patients. Fluid management was targeted to maintain euvolemia. Hypertension was induced in cases of delayed neurological deterioration, defined as new onset neurological deficit or a reduction in the Glasgow coma scale of more than 2 points persisting for more than 1 h [19, 20], with a target mean arterial pressure (MAP) of > 110 mmHg. Mannitol or 5% hypertonic saline solution was only administered in cases of elevated intracranial pressure (ICP > 20 mmHg) as a rescue strategy. Interventional single spasmolysis with papaverine with or without angioplasty was reserved for patients who did not respond to induced hypertension. Patients received minimal amounts of sedatives, such as propofol, midazolam, and dexmedetomidine, which were necessary to prevent ventilator dyssynchrony and patient discomfort. Analgesics, including acetaminophen, nonsteroidal anti-inflammatory medications, and fentanyl, were administered according to sedation and pain scores (Richmond agitation sedation scale and behavior pain scale). Fever, defined as body core temperature > 38.2 °C, was treated with acetaminophen, nonsteroidal anti-inflammatory medications, or cooling devices in ventilated patients. Active maintenance of normothermia with cooling devices was not routinely performed.

### Ventilation

All patients were intubated with a muscle relaxant prior to angiography and initial treatment (surgical clipping or endovascular coiling). The muscle relaxant was discontinued after completion of the initial treatment and was not used as ICU management thereafter. For mechanically ventilated patients, respiratory management involved the use of the spontaneous breathing mode (pressure support mode) with minimal support (i.e., continuous positive airway pressure or a pressure support level ≤ 5 cm H2O). Daily spontaneous breathing trials were not performed. When a patient’s condition stabilized, the patient was weaned off mechanical ventilation and extubated after the completion of the initial treatment.

### Delayed cerebral ischemia

Delayed cerebral ischemia was defined according to the 2010 Multidisciplinary Research Group Definition as the occurrence of secondary neurological deficits or strokes not attributable to another cause. Awake patients with a clinical diagnosis of DCI or patients who required sedation but had suggestive changes in extended neuromonitoring (near-infrared spectroscopy, brain tissue oxygen tension, and cerebral microdialysis) were subjected to radiographic verification. Based on CT angiography and CT perfusion, rescue therapy was initiated [23, 24].

### PaCO_2_

Arterial blood gas (ABG) analyses, including PaCO_2_, were routinely performed at least every 12 h for the first 14 days. The lowest PaCO_2_ value was determined for each 24-h period.

### Body core temperature

Body core temperature was measured continuously using a bladder catheter with a temperature probe. All patients with fever (> 38.2 °C) underwent an infection workup including urine, sputum, blood culture and, in patients with an external ventricular drain, additional cerebrospinal fluid workup. We defined fever days those on which the body temperature could not be reduced below 38.2 °C by fever management.

### Data sampling

The following data were collected from medical charts and the patient data management system: sex, age, Fisher grade, WFNS grade, treatment modality (coiling or clipping), angiographic CVS, occurrence of DCI, ventilatory support (mechanical ventilation, NIV), PaCO_2_ measurements (days < 4.5 kPa, CO_2_ mean, day 0–14), body core temperature in °C (*T*_*mean*_, days > 37.5, days > 38.2, day 0–14), leucocyte count (mean and maximal), CRP level (mean and maximal), functional neurological outcome at hospital discharge (mRS), and hospital mortality. As part of standard treatment, CRP and WBC were measured daily until discharge or at least until day 14. This was not done in less than 10% of cases. The incidence of pneumonia, ventriculitis, and the overall rate of infection were also reported.

### Outcome measures

The primary outcome of our study was the ability of clinical signs of inflammation (hyperventilation, fever) and simple inflammatory laboratory parameters (CRP and WBC) to predict unfavorable outcome at discharge defined mRS > 3. The secondary outcome was the ability of the same variables to predict DCI.

### Statistical analysis

The statistical analyses were performed using the R statistical software version 4.0.2. (www.r-project.org). A two-sided *p* value < 0.05 was considered statistically significant.

To assess the diagnostic value of continuous predictors for a dichotomized mRS (mRS 4–6 versus mRS 0–3), receiver operating characteristic (ROC) curve analysis was performed using the R library “PRROC.” Optimal cutoff values were determined using the Youden’s *J* statistic method. The time course of continuous predictors was assessed by trajectories. To assess the neurological outcome measured by mRS, univariable and multivariable cumulative linkage models were applied using the R library “ordinal.” The multivariable model was complemented by a backward variable selection based on the Akaike’s information criterion (AIC). The predictors were selected according to the literature and considering the univariable results of the ROC analysis and trajectories. They were CVS, DCI, Fisher grade, WFNS scale, age, sex, coiling versus clipping, CRP on day 2 (dichotomized lower vs higher than 100 mg/dl), invasive ventilation longer than 1 day, number of days with fever (*T* > 38.2 °C), and hyperventilation (lowest daily PaCO2 < 4.5 kPa). C-reactive protein at day 2 was chosen because the extent of inflammation at this stage is less likely to be influenced by ICU-acquired infections and may be more representative of the burden of early brain injury caused by the aSAH itself.

To avoid separation, mRS grades 5 and 6, Fisher grades 1 and 2, and WFNS were included with aggregated categories. Because of strong collinearity with CRP, WBC was not included in this model. To further assess the diagnostic value of the predictors for the severity of the neurologic impairment measured by the mRS in the multivariable context and taking into account the ordinal scale of mRS, a random forest analysis was performed using the “cforest” function from the R library “party” to compute the relative variable importance for the prediction [21].

Univariable and multivariable adjusted logistic regressions were performed for the analysis of the secondary outcomes DCI, supplemented by backward variable selection based on AIC. Missing data were corrected using the random forest method.

## Results

### Baseline characteristics of patients

A total of 97 consecutive patients with aSAH admitted to the ICU during the study period were consecutively included in the analysis. Of these, 76 (78%) had a favorable neurological outcome at discharge (mRS 0–3) while 21 (22%) had major disability or died (mRS 4–6). Sixty-three patients (64.9%) were female and 34 (35.1%) were male with a mean age of 58 (SD 13.8) years. Demographic characteristics and treatment modality did not differ significantly between patients with favorable and unfavorable neurological outcome, although patients with unfavorable neurological outcome (mRS 4–6) had more severe clinical and radiological presentation at admission (Table 1). Regarding the secondary outcome, 20 patients (21%) had DCI (Table 2).

**Table 1:** Patient baseline characteristics

	Total	mRS^1^ 0–3	mRS^1^ 4–6	*p* value
Number	97	76	21	
Female	63 (64.9%)	48 (63.2%)	15 (71.4%)	0.482
Mean age (SD^2^)	58.0 (13.2)	57.7 (13.7)	59.2 (11.4)	0.575
WFNS grade				
1–3	58 (59.8%)	52 (68.4%)	6 (28.6%)	0.001
4–5	39 (40.2%)	24 (31.6%)	15 (71.5%)	
Fisher grade				
1–2	17 (17.5%)	17 (22.4%)	0 (0%)	0.010
3–4	80 (82.5%)	59 (77.6%)	21 (100%)	
Treatment				
Coil	44 (45.4%)	34 (44.7%)	10 (47.6%)	0.814
Clip	53 (54.6%)	42 (55.3%)	11 (52.4%)	

^1^Modified Rankin scale

^2^Standard deviation

**Table 2:** Descriptive analysis of patients in dichotomized mRankin scale groups

	Total	mRS^1^ 0–3	mRS^1^ 4–6	*p* value
Number	97	76	21	
Days ICU^2^	11.9 (3.1)	11.7 (3.1)	12.6 (2.8)	0.241
In-hospital mortality	7 (7.2%)	0 (0.0%)	7 (33.3%)	< 0.001
Days mechanical ventilation	3.1 (3.9)	2.0 (3.0)	6.8 (4.7)	< 0.001
Days mandatory ventilation	2.4 (3.0)	1.7 (2.4)	5.1 (3.4)	< 0.001
DCI^3^	20 (20.6%)	9 (11.8%)	11 (52.4%)	< 0.001
Days subfebrile (> 37.5 °C)	6.4 (3.8)	6.1 (3.8)	7.4 (3.7)	0.117
Days febrile (> 38.2 °C)	2.6 (3.0)	2.4 (2.9)	3.7 (3.2)	0.043
Days hyperventilation	11.9 (3.1)	11.6 (3.2)	12.6 (2.8)	0.177
Mean temperature (°C)	37.7 (0.5)	37.6 (0.4)	37.8 (0.5)	0.329
Mean PaCO2 (kPa)	4.5 (0.5)	4.5 (0.5)	4.5 (0.5)	0.514
Mean CRP^4^ (mg/dl)	65.1 (37.5)	56.3 (28.6)	96.6 (48.3)	< 0.001
Infection	18 (18%)	11 (14.5%)	7 (33.3%)	0.069
Pneumonia	10 (10.3%)	5 (6.6%)	5 (23.8%)	0.042
Mean day of diagnosis	5.7 (3.2)	5.4 (2.1)	6.0 (4.2)	0.831
Range day of diagnosis	3.0–13.0	3.0–8.0	3.0–13.0	
Ventriculitis	4 (41%)	3 (3.9%)	1 (4.8%)	0.833

^1^*mRS*, modified Rankin scale

^2^Intensive care unit

^3^Delayed cerebral ischemia

^4^*CRP*, C-reactive protein

### Primary outcome: prediction of unfavorable neurological outcome (mRS 4–6)

In univariable analysis, ischemic complications (OR 3.18, CI 1.37–7.58 for CVS and 10.23, CI 3.42–31.88 for DCI), higher Fisher grade (OR 1.48, CI 0.53–4.20 for grade III and 4.08, CI 1.57–11.0 for grade IV), WFNS grades 4–5 (OR 4.37, CI 2.07–9.52), CRP on the second day after diagnosis (OR 7.14, 3.09–17.27), more than 1 day of invasive mechanical ventilation (OR 11.22, CI 4.95–26.85), and number of days with hyperventilation (OR 1.24 per day, CI 1.10–1.42) and with fever (OR 1.24, CI 1.10–1.40 per day) were associated with worse neurological outcome at discharge as defined by mRS. Sex and treatment modality did not show significant difference in outcome. A tendency for worse outcome with increased age (OR 1.02, CI 1.00–1.05) was observed (Table 3). After multivariate adjustment, ischemic complications (OR 4.49, CI 1.31–15.93 for CVS and OR 18.04, CI 3.68–93.44 for DCI), increased age (OR 1.05, CI 1.01–1.08), and more than 1 day of mechanical ventilation (OR 5.45, CI 1.76–17.40) were significant predictors for a worse neurological outcome. In the model established by a backwards variable selection procedure, a CRP over 100 mg/dl on day 2 after diagnosis (OR 2.70, CI 1.06–6.98) was another independent predictor for worse outcome (Table 3).

**Table 3:** Cumulative link model for prediction of neurological outcome at discharge (mRankin scale)

Variable	Univariable OR^1^	pUni^2^	Multivariable OR^1^	pMulti^3^	Stepwise OR	pStep^4^
Ischemic complications		< 0.001		0.001		< 0.001
CVS^5^	3.18 (1.37–7.58)		4.49 (1.31–15.93)		3.20 (1.27–8.28)	
CVS^5^ + DCI^6^	10.23 (3.42–31.88)		18.04 (3.68–93.44)		9.55 (2.97–32.3)	
Fisher grade		0.005		0.158		
I–II	Reference		Reference		–	–
III	1.48 (0.53–4.20)		0.44 (0.13–1.44)		–	–
IV	4.08 (1.57–11.0)		1.03 (0.33–3.15)		–	–
WFNS^7^ grade		< 0.001		0.919		
1–3	Reference		Reference		–	–
4–5	4.37 (2.07–9.52)		1.06 (0.37–3.04)		–	–
Sex		0.978		0.311		
Female	Reference		Reference		–	–
Male	1.01 (0.48–2.10)		1.57 (0.66–3.80)		–	–
Age (continuous)	1.02 (1.00–1.05)	0.081	1.05 (1.01–1.08)	0.004	1.04 (1.01–1.07)	0.007
Treatment modality		0.902		0.792		
Clip	Reference		Reference		–	–
Coil	0.96 (0.47–1.95)		1.11 (0.50–2.51)		–	–
CRP^8^ day 2		< 0.001		0.052		0.038
≤ 100 mg/dl	Reference		Reference		Reference	
> 100 mg/dl	7.14 (3.09–17.27)		2.56 (0.99–6.73)		2.70 (1.06–6.98)	
Days IMV^9^		< 0.001		0.003		< 0.001
≤ 1	Reference		Reference		Reference	
> 1	11.22 (4.95–26.85)		5.45 (1.76–17.40)		7.07 (2.87–18.23)	
Days hyperventilation (PaCO2 < 4.5 kPa, continuous)	1.24 (1.10–1.42)	< 0.001	0.99 (0.83–1.18)	0.871	–	–
Days fever (*T* ≥ 38.3)	1.24 (1.10–1.40)	< 0.001	1.00 (0.86–1.16)	0.982	–	–
Infection	2.03 (0.81–5.22)	0.132	2.10 (0.68–6.55)	0.196	–	–

^1^*OR*, odds ratio

^2^*pUni*, *p* value univariate

^3^*pMulti*, *p* value multivariate

^4^*pStep*, *p* value stepback analysis

^5^*CVS*, cerebral vasospasm

^6^*DCI*, delayed cerebral ischemia

^7^World Federation of Neurosurgical Societies

^8^*CRP*, C-reactive protein

^9^*IMV*, invasive mechanical ventilation

**Fig. 1:**
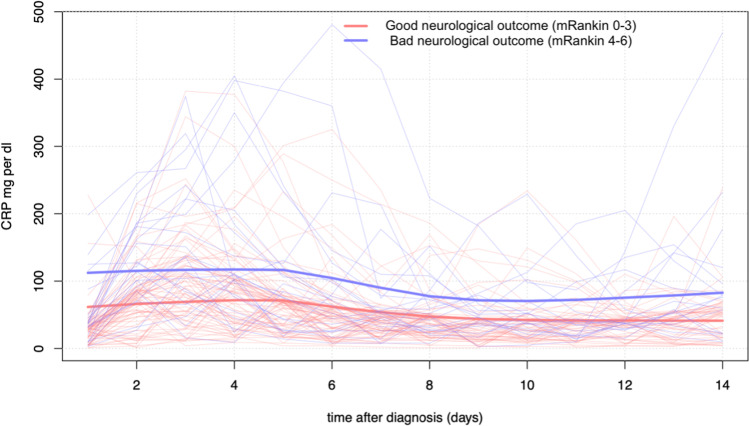
C-reactive protein trajectory over the first 14 days after aSAH in patients with favorable (mRankin scale 0–3) and unfavorable (mRankin scale 4–6) neurological outcome

**Fig. 2:**
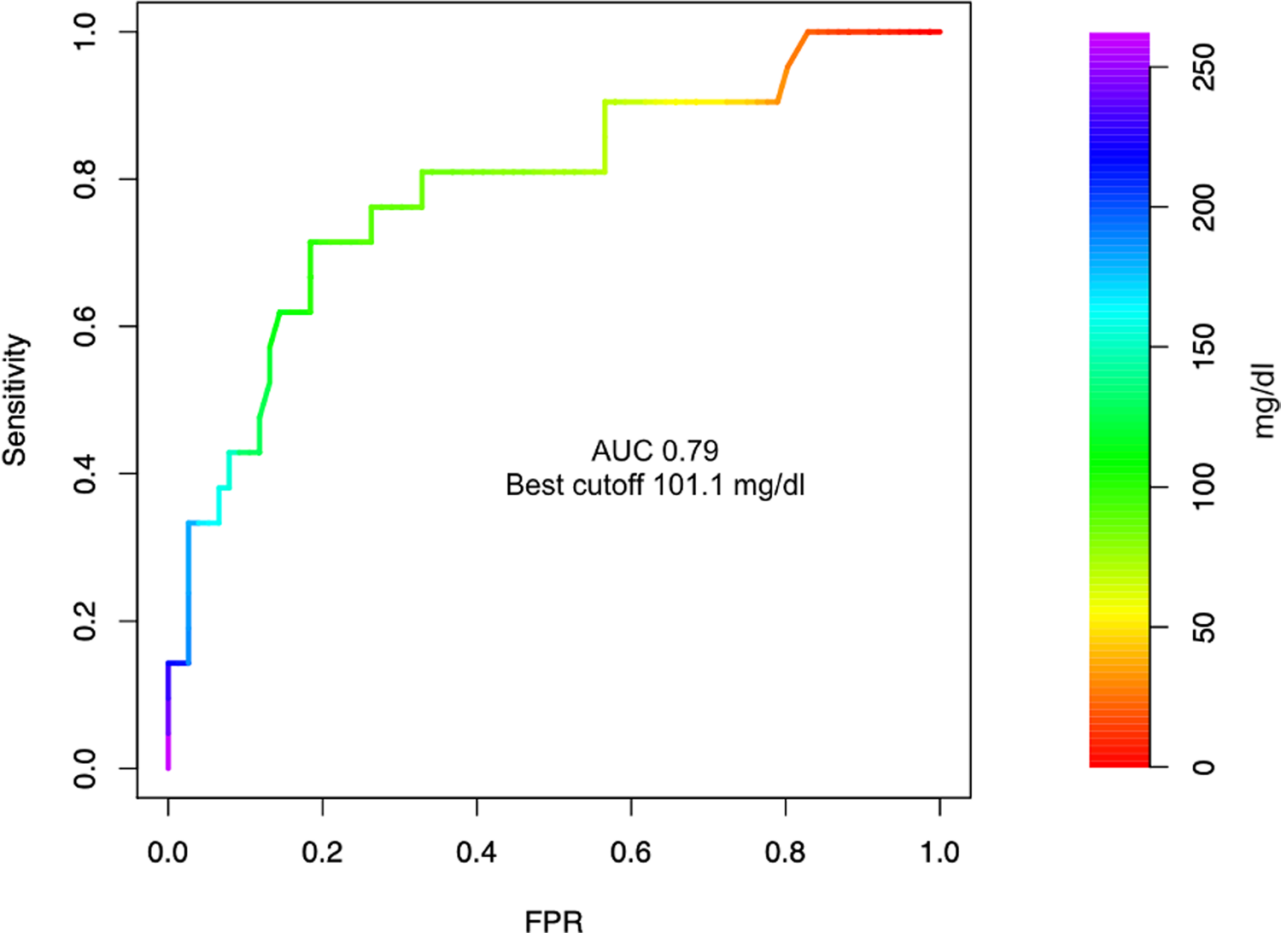
Receiver operating characteristic (ROC) curve analysis of CRP at day 2 as a predictive variable for worse neurological outcome. AUC, area under the curve; FPR, false positive rate

**Fig. 3:**
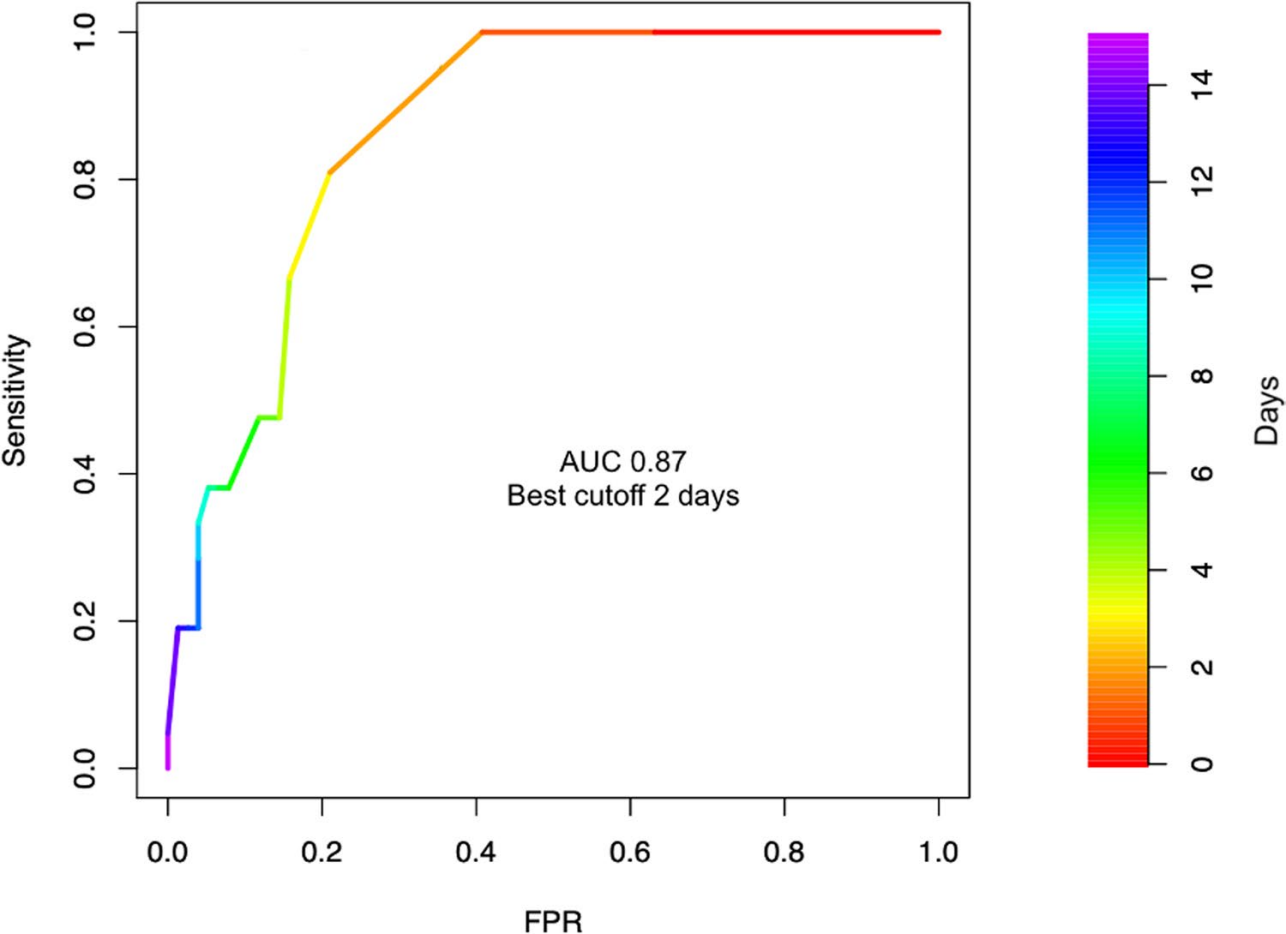
Receiver operating characteristic (ROC) curve analysis of days of mechanical ventilation as a predictive variable for worse neurological outcome. AUC, area under the curve; FPR, false positive rate

Mean CRP values were consistently higher among patients with worse neurological outcome (96.6, SD 48.3 vs 56.3 mg/dl, SD 28.6, *p* < 0.001) for the first 14 days after aSAH (Fig. 1). When dichotomizing the outcome (mRS 0 to 3 versus 4 to 6), receiver operating characteristic (ROC) curve analysis revealed the highest diagnostic value for CRP on day 2 (AUC 0.79, CI 0.67–0.91) and days with mechanical ventilation (AUC of 0.87, CI 0.80–0.94) (Figs. 2 and 3). Mean WBC was also higher in patients with mRS 4–6 at discharge (12.0, SD 2.3 vs 10.6 G/l, SD 2.2, *p* = 0.019), while there was no statistically significant difference in mean PaCO_2_ (4.5, SD 0.5 vs 4.5, SD 0.5 kPa, *p* = 0.054) and mean body temperature (37.8, SD 0.5 vs 37.6 °C, SD 0.4, *p* = 0.329) between the groups. The incidence of pneumonia was higher in patients with worse neurological outcome (6.6% vs 23.8%, *p* = 0.042), while the overall occurrence of infection barely reached statistical significance (14.5% vs 33.3%, *p* = 0.069). However, no infection was observed before day 3 after aSAH (Table 2).

### Secondary outcome: prediction of delayed cerebral ischemia

In univariable and multivariable analysis, the only predictor for DCI was the number of days of hyperventilation (OR 1.32, CI 1.02–1.87) (Table 4).

**Table 4:** Logistic regression for prediction of delayed cerebral ischemia

Variable	Univariable OR^1^	pUni^2^	Multivariable OR^1^	pMulti^3^	Stepwise OR^1^	pStep^4^
Fisher grade		0.065		0.327		–
I–II	Reference		Reference		–	
III–IV	4.98 (0.92–92.85)		2.86 (0.39–59.05)		–	
WFNS^5^ grade		0.625		0.799		–
1–3	Reference		Reference		–	
4–5	1.28 (0.47–3.47)		1.17 (0.35–3.83)		–	
Gender		0.592		0.315		–
Female	Reference		Reference		–	
Male	0.75 (0.24–2.10)		0.53 (0.14–1.79)		–	
Age (continuous)	0.98 (0.94–1.01)	0.218	0.97 (0.93–1.01)	0.186	–	–
Treatment modality		0.332		0.625		
Clip	Reference		Reference		–	–
Coil	0.61 (0.22–1.65)		0.75 (0.23–2.38)			
Days hyperventilation (PaCO2 < 4.5 kPa, continuous)	1.36 (1.08–1.90)	0.005	1.32 (1.02–1.87)	0.031	1.40 (1.12–1.97)	0.002
Days fever (*T* ≥ 38.3)	1.13 (0.96–1.31)	0.139	1.06 (0.87–1.30)	0.546	–	–
CRP^6^ day 2		0.661		0.843	–	–
≤ 100 mg/dl	Reference		Reference			
> 100 mg/dl	1.26 (0.43–3.52)		1.14 (0.32–3.97)			
Infection	0.19 (0.01–1.00)	0.050	0.11 (0.01–0.67)	0.014	–	–

^1^*OR*, odds ratio

^2^*pUni*, *p* value univariate

^3^*pMulti*, *p* value multivariate

^4^*pStep*, *p* value stepback analysis

^5^World Federation of Neurosurgical Societies

^6^*CRP*, C-reactive protein

## Discussion

**Fig. 4:**
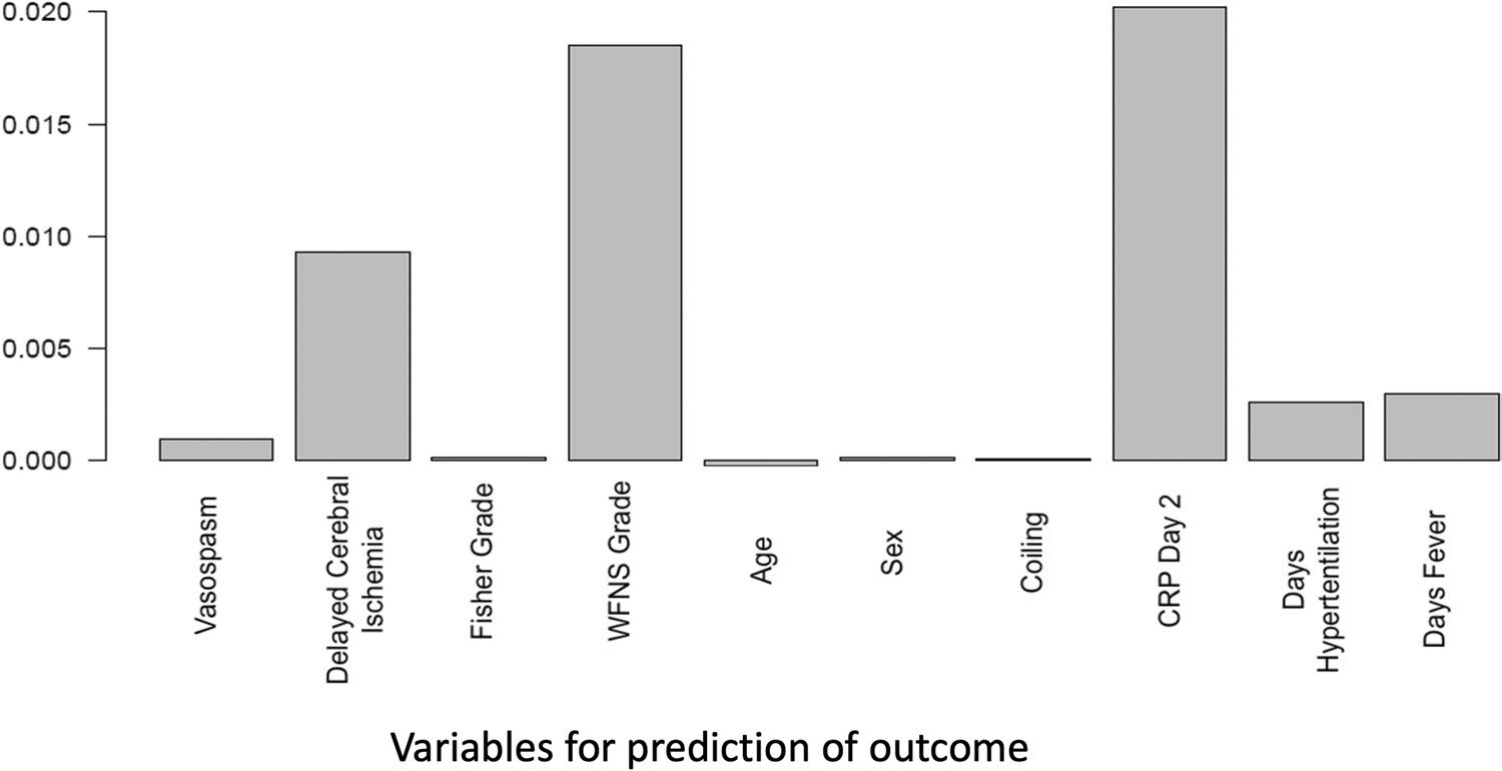
Relative importance of single variables in the prediction of higher modified Rankin scale

In our study, early elevated CRP levels along with ischemic complications such as DCI, increased age, and more than 1 day of mechanical ventilation were significant predictors of worse neurological outcome at hospital discharge. Despite a significant improvement in outcomes, aSAH still bears high levels of morbidity and mortality [13, 25]. Easily obtainable early predictors of clinical deterioration can help guide therapy, monitoring, and diagnostic procedures in these patients. In our analysis, a serum CRP level greater than 100 mg/l on the second day of hospitalization was the inflammatory marker with the strongest independent correlation with a worse neurological outcome (Fig. 4). Serum CRP on day 2 also had the highest diagnostic discriminatory power for worse neurological outcome in the ROC curve analysis AUC of 0.79. Fever and hyperventilation were also associated with a worse neurological outcome, but were not predictive of higher mRS after multivariate adjustment. Moreover, mean pCO2 and temperature did not differ between the dichotomized mRS groups. However, consistent with previous studies the number of days of hyperventilation was an independent predictor of DCI [19, 26, 27]. As expected, duration of mechanical ventilation was also associated with worse neurological outcome as it represents a surrogate of disease severity (Fig. 3).

Our findings are consistent with previous reports of Jeon et al., who described an association between CRP on the first 2 days after securing of the aneurysm and the occurrence of unfavorable outcome, while using a lower cutoff of 40 mg/l [10]. Similar findings have also been described in a post hoc analysis of the STASH trial, where CRP was an early independent predictor of poor outcome, even in patients with good radiological and clinical grading at presentation [22]. Other studies found an association between elevated CRP in the early phase after aSAH and higher disability scores but failed to show that it was an independent predictor of worse outcome [8, 11, 15]. Muroi et al. also found an association between elevated CRP and unfavorable neurological outcome and observed higher CRP levels in these patients, especially between days 8 and 14 after admission. However, in contrast to our results, they found that CRP was not an independent predictor of neurological outcome after correction for concomitant infection [14]. A possible explanation for this difference is the overall much higher CRP level in our patient cohort, especially in the first week after admission, which may reflect later hospital presentation. Our findings support the hypothesis that early systemic inflammation may play an important role in the pathogenesis of brain injury in aSAH. Interestingly found no significant association between CRP levels and DCI. We believe that this discrepancy may reflect other mechanisms of early brain injury that play a role in the early phase after aSAH such as transient global ischemia and intracranial pressure elevation after aneurysmal rupture, neuroinflammation, dysfunction of the blood–brain barrier as well as cortical spreading depolarizations [2]. We acknowledge that, because of its lack of specificity, serum CRP may be influenced by infection and sepsis in an ICU population. However, in our analysis the best predictor of neurological outcome among inflammatory markers and signs was the CRP level on the second day of hospitalization which is unlikely to be influenced by infections because these classically occur later in the course of the disease. Furthermore, no episode of infection, including pneumonia, was diagnosed or treated before day 3 in our patient population. We believe that our results provide further evidence that CRP may serve as an early predictor of clinical deterioration, independent of the initial radiologic and clinical grading of aSAH and the occurrence of ischemic complications. Further research is needed to determine whether patients with higher CRP levels might benefit from more intensive monitoring for deterioration and whether a correlation between systemic inflammation and aSAH morbidity exists.

## Limitations

Some data, such as body temperature, may have been influenced by the use of analgesics with antipyretic effects and targeted temperature management, while pCO2 levels may have been confounded by controlled mechanical ventilation, as patients with a higher modified Rankin score had more days of mandatory mechanical ventilation (5.1 vs 1.7), making spontaneous hyperventilation impossible. Neurological outcome at discharge may represent a relative short follow-up time. In addition, the CRP cutoff of 100 mg/dl was retrospectively determined by optimizing the Youden’s analysis of ROC data and the choice of day 2 was arbitrary to minimize the influence of ICU-acquired infections. Another limitation of our study is the absence of precise data reporting the frequency and technique of salvage spasmolysis in patients’ refractory to induced hypertension. An additional limitation is the absence of modified Fisher scale data in our analyses; this could have been a potentially better association on the risk of DCI. Finally, we found inconsistent signals concerning the association of infection with DCI and outcome; however, the analysis of this effect was not the objective of our analysis and should be investigated in a larger cohort.

## Conclusion

Early elevated CRP levels and ischemic complications such as DCI (OR 3.54 for CVS and 12.10 for DCI), older age, and more than 1 day of mechanical ventilation were significant predictors for a worse functional neurological outcome at hospital discharge. Clinicians should be aware that a high CRP level may be a marker of subsequent deterioration even in patients presenting with good grade aSAH.
